# Improving the specificity of exon prediction using comparative genomics

**DOI:** 10.1186/1471-2164-9-S2-S13

**Published:** 2008-09-16

**Authors:** Jing Wu

**Affiliations:** 1 Department of Statistics, Purdue University, 150 N. University Street, West Lafayette, IN 47906, USA

## Abstract

**Background:**

Computational gene prediction tools routinely generate large volumes of predicted coding exons (*putative exons*). One common limitation of these tools is the relatively low specificity due to the large amount of non-coding regions.

**Methods:**

A statistical approach is developed that largely improves the gene prediction specificity. The key idea is to utilize the evolutionary conservation principle relative to the coding exons. By first exploiting the homology between genomes of two related species, a probability model for the evolutionary conservation pattern of codons across different genomes is developed. A probability model for the dependency between adjacent codons/triplets is added to differentiate coding exons and random sequences. Finally, the log odds ratio is developed to classify putative exons into the group of coding exons and the group of non-coding regions.

**Results:**

The method was tested on pre-aligned human-mouse sequences where the putative exons are predicted by GENSCAN and TWINSCAN. The proposed method is able to improve the exon specificity by 73% and 32% respectively, while the loss of the sensitivity ≤ 1%. The method also keeps 98% of RefSeq gene structures that are correctly predicted by TWINSCAN when removing 26% of predicted genes that are in non-coding regions. The estimated number of true exons in TWINSCAN's predictions is 157,070. The results and the executable codes can be downloaded from

**Conclusion:**

The proposed method demonstrates an application of the evolutionary conservation principle to coding exons. It is a complementary method which can be used as an additional criteria to refine many existing gene predictions.

## Background

One of the most important challenges in gene prediction is to identify relatively small amounts of coding DNA among a large number of DNA sequences. Computational approaches based on single genomes, including the identification of likely splice sites [1], integrated models [2], and hidden Markov models such as GENSCAN [3] have been developed to identify a large number of genes [4]. In addition to these approaches, *sequence homology *– or comparative genomics – has been employed [5,6]. Instead of using a single genome to predict gene structures, sequence homology uses a pair or multiple DNA sequences of related species (e.g., human and mouse) to study the conservation across them [7,8]. Recently, a new class of gene-prediction algorithms which exploits the power of comparative genomics have been developed, these include but are not limited to ROSETTA [9], CEM [10], TWINSCAN [11], SLAM [12], and SGP2 [13]. Relative to the single genome approaches, these programs have substantially reduced the number of false predictions, (i.e. improved specificity), but the reduction is not enough simply because of the large amount of non-coding DNA.

A high specificity in exon prediction is shown by Nekrutenko et al. [14] when the exon location and frame are given. Nekrutenko et al. [14] used the *K*_*A*_/*K*_*S *_ratio test to detect the difference between the conservation of codons and non-codons, where *K*_*S *_and *K*_*A *_denote the synonymous and non-synonymous substitutions, respectively. Wu and Haussler [15] incorporated the log odds scores of codons in a hidden markov model that predicts exon structures. Similar as in [16], the log odds ratio in [15] was used to indicate the existence of an exon in the aligned sequence segments without the knowledge of the exon structure. These methods demonstrate that log odds ratios based on codon conservation can be used to indicate the possibility of a coding region existing in the alignment of human and mouse sequences [15,16]. However, since exon structure was assumed unknown, the high accuracy shown in [14] was not attained.

Existing methods that predict gene structures (using homology or single genomes) have identified many candidates exons, (*putative exons*), e.g. exons predicted by GENSCAN, TWINSCAN etc., which bridge the power of comparative genomic approaches [14-16] and existing gene structure prediction methods [9,11-13]. Since the putative exons naturally split into three types: those that exactly overlap with coding exons, those that partly overlap with coding exons, and those that totally fall into the non-coding regions, if one can develop a methodology which effectively identifies putative exons of third type – those contain nothing but non-coding DNA – one then can filter them out and largely improve the specificity.

The proposed methodology is a scoring method that is based on the idea of homology which takes advantage of the conservation law between two related species. Given that two sequences of related species (e.g., human and mouse) have been aligned, for each codon or triplet in the non-coding region, a probability model is developed for their dependency on the adjacent codon/triplet in the same sequence, as well as their conservation across different sequences (e.g., human and mouse). Based on such models, it is possible to calculate, for each putative exon, the likelihood based on codons or triplets from non-coding regions, or equivalently the log odds ratio. Intuitively, the larger the log odds ratio, the more likely the putative exon is comprised of codons, and vice versa. Therefore, typically the putative exons that contain only non-coding DNA should have a relatively smaller log odds ratio.

Compared to the existing methods, the probability model is equivalent to the model in [14] except for the codon dependency model. However, an important difference is that it provided an application for the homology approach studied by [14], as it directly implements the log odds ratio methodology to the putative exons identified by other programs (e.g., GENSCAN and TWINSCAN).

## Results

The proposed method is first compared with GENSCAN, TWINSCAN, and *shorthmm *in [15]. In order to train and test the proposed method, the data sets used in [15] were adopted. Then, the effects of the proposed method on TWINSCAN's prediction of the entire RefSeq exons and RefSeq genes after filtering out the false predictioins are examined.

### Data sets

The test data are summarized in Table 1. First, the sets of clearly orthologous exons and potential non-exons used in [15] were used to compare the proposed method with other methods. In [15], the locations of RefSeq exons [17-19] were first downloaded from assembly hg12 (June 2002) in UCSC's genome database [20]. The non-overlapping RefSeq exons were then extended 90 bps on both ends and the human-mouse alignments [21] of the extended exons were extracted from the chained and netted human-mouse alignments in assembly hg12. Those RefSeq exons that have full extended alignments were called the clearly orthologous exons. The potential non-exons in human genome were obtained by eliminating the alignment segment of human mRNAs and ESTs and the 100 bps beyond their end points from the chained and netted human-mouse alignments in assembly hg12, where the coordinates of human mRNAs and ESTs were based on the annotations of mRNA and ESTs in assembly hg12. Out of the two sets, 5,000 alignments of clearly orthologous exons and 20,000 alignments of potential-non exons were used as training data and the rest were used as test data in [15]. In this experiment, the coordinates of the clearly orthologous exons and the potential non-exons were first lifted from hg12 to the assembly of hg17 in UCSC's genome database by the batch coordinate conversion [22]. The alignment of clearly orthologous exons and potential non-exons were then extracted from the chained and netted alignments with mouse [23] and dog [24] in assembly hg17 in UCSC's genome database (mm5, May 2004; v. 1.0., July 2004; *axtNet *folder) respectively. The clearly orthologous exons that do not have full alignments were discarded. In the remaining clearly orthologous exons and potential non-exons, those used as training sets in [15] were used to train the proposed model and the rest alignments were used to test the model. The coordinates of the sequences can be downloaded from .

**Table 1:** Summary of the data sets.

	clearly orthologous exons (TP)	potential non-exons (FP)	potential non-genes (FP)	RefSeq exons (TP)	RefSeq genes (TP)
size	76,229 (1.2 × 10^7 ^bps)	1,518,082 (8.3 × 10^8 ^bps)	--	172,042 (2.9 × 10^7^)	20,193
GENSCAN	--	--	--	117,860	3,497
TWINSCAN	--	--	--	118,650	5,131
GENSCAN (w/mouse)	53,217	54,360	4,856	115,551	3,284
TWINSCAN (w/mouse)	54,879	12,276	1,172	117,100	4,944
GENSCAN (w/dog)	52,712	49,899	--	--	--
TWINSCAN (w/dog)	54,257	11,095	--	--	--

The first row lists the type of sequences in the data set. The second row lists the number of the sequences in each type and the corresponding base pairs. The row of GENSCAN lists the number of exons predicted by GENSCAN with both ends matching RefSeq exons, the number of genes predicted by GENSCAN that exactly match RefSeq genes. The row of GENSCAN (w/mouse) lists the number of exons predicted by GENSCAN, which have full alignments with mouse, with both ends matching clearly orthologous exons, the number of the predicted exons, which have full alignments with mouse, with both ends within or matching potential non-exons, and the number of genes predicted by GENSCAN, which have full alignments with mouse, having all exons being in potential non-exons. The row of GENSCAN (w/dog) lists the number of exons predicted by GENSCAN, which have full alignments with dog, with both ends matching clearly orthologous exons and the number of the predicted exons, which have full alignments with dog, with both ends within or matching potential non-exons. The row of TWINSCAN, TWINSCAN (w/mouse), and TWINSCAN (w/dog) list the number of exons and genes collected the same way as those related to GENSCAN from TWINSCAN's prediction.

Next, the putative exons of GENSCAN and TWINSCAN were downloaded from assembly hg17. The alignments of putative exons were also extracted from the chained and netted alignments of human-mouse and human-dog in assembly hg17.

Last, to examine the effect of the proposed method on the correctly predicted RefSeq exons and RefSeq genes, the locations of the entire 172,042 non-overlapping human RefSeq exons were downloaded from UCSC's genome database (hg17), which correspond to 20,193 RefSeq genes.

The true positive and false positive are defined as follows. A putative exon is called a true positive (TP) when both ends of the putative exon match a clearly orthologous exon. A putative exon is called a false positive (FP) when both ends of the predicted exon are within or match a potential non-exon. A putative gene is called a true positive when the gene exactly matches a RefSeq gene. A putative gene is called a *potential non-gene *(FP) when all the exons of the gene are located in potential non-exons.

### Application of the log-odds score on the existing algorithms

To illustrate the improvement of existing gene prediction methods based on single species, the improvement of GENSCAN's predictions using the log odds ratio is compared with TWINSCAN's predictions, where TWINSCAN is an improvement relative to GENSCAN by incorporating conservation information not only in exon models but also in other parts of the gene structure (e.g. splicing sites etc.) to GENSCAN. Another comparison is between TWINSCAN's predictions that have high log odds ratios and the predictions from [15], where [15] incorporated the same probability matrices as in the log odds ratio into a model that predicts exon structure. The results are summarized in Table 2, which show that the refinement of GENSCAN's predictions gives comparable results to TWINSCAN's and the refinement of TWINSCAN's prediction gives comparable results to [15]. These comparisons show that one could gain similar improvements in false positive rate by merely refining the existing results instead of refining the original prediction model. A set of simulated alignments, i.e. 26 alignments with length around 100,000 bps are also generated according to the frequency and the conservation of the nucleotides in the alignment of potential non-exons. To study the threshold, we ran GENSCAN on the simulated data and obtained 47 false predictions. By setting the threshold for the log odds ratio of the alignments that are falsely predicted at -0.33, only 3 false predictions remains, corresponding to a 94% improvement in false positive rate.

**Table 2:** Comparing the enhancement on putative exons with existing models results based on human-mouse sequence conservation.

	clearly orthologous exons (TP)	potential non-exons (FP)
size	76,229	1, 518, 082
GENSCAN (w/mouse)	53,217 (69.8%)	54,360 (3.58%)
GENSCAN (w/mouse) *S *> -0.33	52,682 (69.1%)	14,604 (0.95%)
TWINSCAN (w/mouse)	54,879 (72.0%)	12,276 (0.8%)
TWINSCAN (w/mouse) *S *> -0.12	54,331 (71.3%)	7,876 (0.5%)
*shortHMM S *> 0.69 (w/mouse)	74.5%	0.77%

The number of clearly orthologous exons and potential non-exons in the test set are listed in the row of size. The rows of GENSCAN and TWINSCAN list the numbers of putative exons provided by GENSCAN and TWINSCAN respectively. The thresholds for GENSCAN and TWINSCAN are set so that 99% of the correct predictions of GENSCAN and TWINSCAN that have alignments are kept. The percentages in the parentheses are the true positive and false positive rates relative to the sizes of the test sets. The row of *shortHMM *is cited from [15].

The effect on all RefSeq exons and RefSeq genes that are correctly predicted by TWINSCAN when thresholding on the log odds ratios are listed in Table 3 and Table 4, which show that by losing 2% of correctly predicted RefSeq exons, thresholding on putative exons could remove 37% false exons from TWINSCAN's predictions and by losing 2% of correctly predicted RefSeq genes, thresholding on putative exons could remove 26% false genes from TWINSCAN's predictions.

**Table 3:** Improvement of putative exons from TWINSCAN.

	RefSeq exons (TP)	potential non-exons (FP)
size	172,042	1, 518, 082
TWINSCAN	118,650 (69.0%)	12,276 (0.8%)
TWINSCAN (w/mouse) *S *> -0.12	115,909 (67.1%)	7,876 (0.5%)

Results based on human-mouse conservation. The number of RefSeq exons and potential non-exons in the test set are listed in the row of size. The row of TWINSCAN lists the number of putative exons provided by TWINSCAN. The threshold for TWINSCAN is set so that 99% of the correct predictions of TWINSCAN that have alignments are kept. The percentages in the parentheses are the true positive and false positive rates relative to the size of the test set.

**Table 4:** Improvement of putative genes from TWINSCAN.

	RefSeq genes (TP)	potential non-genes (FP)
size	20,193	--
TWINSCAN	5,131	1,172
TWINSCAN (w/mouse) *S *> -0.9	4,826	870

Results based on human-mouse conservation. The number of RefSeq genes is listed in the row of size. The row of TWINSCAN lists the number of putative genes provided by TWINSCAN. The threshold for TWINSCAN is set so that 98% of the corrected predicted genes of TWINSCAN are kept.

**Figure 1:**
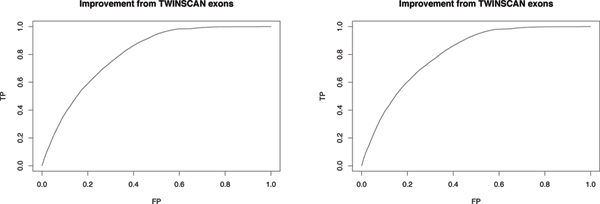
**Improving TWINSCAN's prediction on exons**. ROC curves by applying the log odds ratio on TWINSCAN's exons. The x-axis is the false prediction rate (FP) of the exon by the log odds score and the y-axis is the true prediction rate (TP) of the exon by the log odds score. The upper graph is the result from human-mouse alignments of TWINSCAN's exons. The lower graph is the result from human-dog alignments of TWINSCAN's exons. The plot shows that by using the log odds score to refine TWINSCAN, we could largely reduce the number of false predictions, e.g., by 32% while keeping over 99% of true positives. The plot also shows that the improvement on TWINSCAN is not affected by the type of alignments used since the two curves are almost identical.

The overall refinements on exon prediction by thresholding of the log odds score of TWINSCAN's exons in clearly orthologous exons and potential non-exons are shown by the ROC curve in Figure 1. In the ROC curve, for a given threshold on the log odds ratio, TP is the fraction of the true exon from TWINSCAN with the log odds score greater than the threshold and FP is the fraction of the false exon from TWINSCAN with the log odds score greater than the threshold. The figures show that the level of refinement is almost identical for human-mouse and human-dog alignments.

### Whole genome scan

Using the human-mouse alignments, the entire 182,412 exons predicted by TWINSCAN are scored. By setting the threshold at -0.12 as in Table 2 and Table 3, the estimated number of true exons in TWINSCAN's predictions is 157,070. The scores can be downloaded from .

## Discussion

This paper demonstrates the application of the scoring method as an addition to existing gene prediction methods. The scoring method efficiently removes conserved non-coding regions from putative exons. The improvement over GENSCAN and TWINSCAN demonstrates that the method can not only benefit prediction methods based on a single organism, but also benefit prediction methods based on comparative genomics. The notable improvement in TWINSCAN's sensitivity is especially encouraging since TWINSCAN also incorporated homology in its algorithm. Furthermore, the application of the proposed scoring method is not limited by the availability of alignments, since more than 98% of the total RefSeq exons predicted by GENSCAN and TWINSCAN have full alignments with mouse and dog sequences.

The proposed scoring method considers a first order dependency in the codons. Because of the large parameter space brought by the dependency model, directly extend the current model to a higher order dependency model would make the estimation of the parameters less accurate.

One limitation of the proposed approach is that it does not predict new exons. Specifically, the performance of the method is dependent on the gene prediction method that provides putative exons. The sensitivity of the proposed scoring method is bounded by the sensitivity of the method that provides the putative exon. As shown in the results, in order to filter out false predictions, some sacrifice, i.e. 1% – 2%, in sensitivity is necessary. Although the improvement in specificity is not sensitive to the alignment used, it does depend on how the putative exon is obtained (e.g., from a single organism or from two related genomes). The level of the improvement of an existing method depends on how much of the conservation information used in the proposed scoring method has already been used to generate the putative exons.

For example, post-processing the predictions reported by Wu and Haussler [15] would not remove any false predictions, since Wu and Haussler [15] incorporated the same probability matrices as in this paper in their hidden Markov model.

## Conclusion

The proposed scoring method illustrates a strategy of data refinement. By examining the difference between the conservation and dependency between the codon and the triplet in the alignment, it conducts a filtering process on gene prediction results. The benefit of this approach is that it can be used as an addition to existing algorithms that predict gene structures to improve prediction quality.

## Methods

### Hypothesis testing

For a set of putative exons, a log odds score for each individual is developed. We test whether a putative exon is a coding exon or not based on the log odds ratio. Our hypotheses for each putative exon are:

$$\begin{array}{l} H_{0}:\text{the putative exon is from a non-coding region};\\ H_{1}:\text{the putative exon is a coding exon}. \end{array}$$

The proposed scoring method is based on the principle that functional elements such as exons tend to be more strongly conserved through evolution than random genomic sequences, and adjacent codons tend to depend on each other. A log-odds ratio is developed to capture this information. In detail, call the genomic sequence of interest, the *target sequence*, and the sequence from a related species that is aligned to the target sequence, the *information sequence*. Let *X *= {*h*_1_,..., *h*_*n*_} be a putative exon made of *n *codons, with partial codons on both ends and stop codons removed. Let *m*_*i *_be the triplet in the information sequence aligned to *h*_*i*_, *i *= 1,..., *n*. For each *X*, the log odds ratio is defined as follows,

(1) $$S=\frac{1}{n-1}\log\frac{P_{A}(h_{2}|h_{1})P_{B}(m_{2}|h_{2})\cdots P_{A}(h_{n}|h_{n-1})P_{B}(m_{n}|h_{n})}{Q_{A}(h_{2}|h_{1})Q_{B}(m_{2}|h_{2})\cdots Q_{A}(h_{n}|h_{n-1})Q_{B}(m_{n}|h_{n})},$$

where the probability matrix *P*_*A *_gives the conditional probability of observing codon *h*_*i *_given the previous codon is *h*_*i*-1_, *P*_*B *_gives the conditional probability of observing a triplet *m*_*i *_given *h*_*i *_is a codon, *Q*_*A *_gives the conditional probability of observing a triplet *h*_*i *_from non-coding regions given the previous triplet is *h*_*i*-1_, *Q*_*B *_gives the conditional probability of observing a triplet *m*_*i *_given *h*_*i *_is from non-coding regions.

The hypothesis testing is performed by thresholding the log odds score. That is, given a cutoff value *t*, we accept *H*_1 _if and only if *S *> *t*. Hence, we predict that a putative exon is an exon when *S *> *t *and it is not an exon when *S *≤ *t*. If the putative exon does not have enough base pairs in the alignment to be scored, we accept *H*_0_. For a putative gene, we predict it is a gene if and only if we accept *H*_1 _for all the exons in the gene.

### Training the model

The method is trained and tested on human-mouse and human-dog sequence alignments. Since the estimation procedure on the two types of alignments are equivalent, only the estimation of the probability matrices in definition (1) from the training sets of human-mouse sequence alignments is introduced.

Specifically,

(2) $$P_{A}(h|h^{\prime })=\frac{\text{Number of pairs }(h^{\prime }h)+e}{\text{Number of }h^{\prime }+125e},$$

(3) $$P_{B}(m|h)=\frac{\text{Number of pairs }(hm)+e}{\text{Number of }h+125e},$$

(4) $$Q_{A}(a|a^{\prime })=\frac{\text{Number of pairs }(a^{\prime }a)+e}{\text{Number of }a^{\prime }+125e},$$

(5) $$Q_{B}(b|a)=\frac{\text{Number of pairs }(ab)+e}{\text{Number of }a+125e},$$

where *e *= 1 is the pseudo-count added, *h *is a codon in clearly orthologous exons, *h' *is the codon before *h*, *m *is the triplet aligned to *h*, *a *is a triplet in potential non-exons, *a' *is the triplet before *a*, and *b *is the triplet aligned to *a*. The probability matrices can be downloaded from , where for any two nucleotide triplets *c*_1_*c*_2_*c*_3 _and *d*_1_*d*_2_*d*_3_, *c*_*k*_, *d*_*k *_∈ {*A*, *C*, *G*, *T*, *indel*}, the nucleotides are coded as *A *= 0, *T *= 1, *G *= 2, *C *= 3, indel = 4, *P*(*d*_1_*d*_2_*d*_3 _| *c*_1_*c*_2_*c*_3_) corresponds to the (*i*, *j*)-th entry *i *= 25*c*_1 _+ 5*c*_2 _+ *c*_3_, *j *= 25*d*_1 _+ 5*d*_2 _+ *d*_3_.

## Competing interests

The author declares that they have no competing interests.
